# Do more with Less: Improving High Parameter Cytometry Through Overnight Staining

**DOI:** 10.1002/cpz1.589

**Published:** 2022-11-14

**Authors:** Carly E. Whyte, Damon J. Tumes, Adrian Liston, Oliver T. Burton

**Affiliations:** ^1^ Centre for Cancer Biology SA Pathology and University of South Australia Adelaide Australia; ^2^ Immunology Programme Babraham Institute Cambridge United Kingdom

**Keywords:** antibody incubation, high‐parameter cytometry, optimization, overnight staining, spectral cytometry titration

## Abstract

Recent advances in flow cytometry have allowed high‐dimensional characterization of biological phenomena, enabling breakthroughs in a multitude of fields. Despite the appreciation of the unique properties of antigens and fluorophores in high‐parameter panel design, staining conditions are often standardized for short surface stains, regardless of antibody affinity or antigen accessibility. Here, we demonstrate how increasing antibody incubation times can lead to substantial improvements in sensitivity, maintaining specificity, and reducing background, while also significantly reducing the costs of high‐parameter cytometry panels. Furthermore, overnight staining reduces the influence of interexperimental variability, assisting accurate pooling over experiments over extended time courses. We provide guidance on how to optimize staining conditions for diverse antigens, including how different fixation strategies can affect epitope accessibility. Overnight staining can thus substantially improve the resolution, repeatability, and cost‐effectiveness of high‐parameter cytometry. © 2022 The Authors. Current Protocols published by Wiley Periodicals LLC.

## INTRODUCTION

Flow cytometry is a foundational single‐cell technique used to characterize diverse biological systems. Over the last decades, our increased understanding of the complexity of biological systems has pushed the need to expand flow cytometry capabilities to simultaneously characterize increasing numbers of parameters. Technological innovations such as additional lasers and unique fluorophores coupled with improvements in compensation (Roca, Burton, Gergelits et al., 2021) and the advent of spectral cytometry have only accelerated the demand and appreciation for high‐parameter cytometry.

High‐parameter flow panels require careful design in order to successfully analyze each antigen (Ferrer‐Font et al., 2021). The biological characteristics of the antigen must be fully considered, including the localization and level of expression on cell types of interest. General design rules consider antigen abundance, with weakly expressed antigens often paired with bright fluorophores, and strongly expressed antigens detected with dimmer fluorophores (Mahnke & Roederer, 2007). The characteristics of each fluorophore also need to be carefully considered in order to limit the effects of spectral spread into channels that require more precise resolution. Even with careful panel design, many difficulties can limit the performance of high‐parameter flow panels. Fluorophore choice for a given antibody is often limited by the commercial availability of conjugated clones. The affinity of the antibody clone for its target is another limitation, with lower affinity clones only able to be appropriately resolved when conjugated to bright fluorophores. Biological barriers also exist, where stimulation of cells (whether *in vivo* or *ex vivo*) can lead to downregulation or internalization of surface antigens, making it difficult to accurately resolve the level of expression by the cell. All of these issues can limit which antibodies can be used for flow panels and lead to sub‐optimal resolution of antigens. Compromises must often be made in the quest to balance spectral spread with successful detection of expression for an ideal panel of antigens. This is particularly critical with the increasing number of parameters being measured simultaneously, with 40+ fluorophores being routinely investigated by spectral cytometry.

Despite the unique complexity of each antigen and antibody‐fluorophore conjugate being a key consideration for panel design, the actual staining conditions are often standardized across diverse panels, with many antigens detected by short, 30‐min incubation times with antibodies on the surface of unfixed cells. This can be sufficient for many antigens, particularly those that are highly expressed, detected by high‐affinity antibodies, or coupled with bright fluorophores. However, published flow cytometry data all too often have poorly discriminated positive signals, leading to discrepancies in setting gates and subsequent inaccurate quantitation of cells and marker expression. Vast improvements in the quality of staining can be achieved by optimizing the staining conditions for each antigen. In this article, we discuss how optimization of staining conditions, particularly related to antibody incubation times, can improve the accuracy of data obtained by flow cytometry. In particular, we emphasize the addition of overnight antibody staining to your flow staining arsenal to improve the discrimination of cell lineages and marker expression. We discuss the benefits of overnight staining with regard to sensitivity, accuracy, reproducibility, cost, and flexibility. We also describe the considerations and limitations that need to be contemplated when using and optimizing overnight staining.

## IMPROVING FLOW STAINING WITH INCREASED INCUBATION TIMES

One aspect of critical importance in improving the resolution of antibody binding is the incubation time of the antibody with its antigen target. Antibody binding is a reversible reaction driven by non‐covalent bonds, including hydrogen, hydrophobic, electrostatic, and van der Waals bonds (Reverberi & Reverberi, 2007). Initially, the rate of antibody binding to its antigen is greater than the dissociation of the antibody:antigen complex. This is true until the point of equilibrium, where the rate of association and dissociation are equal. In routine flow cytometry, antibodies are mostly incubated for 15 to 60 min at relatively high concentrations (μg/ml). As antibody binding follows a non‐linear sigmoidal curve, substantial binding occurs rapidly, within minutes. However, similar levels of binding can be achieved with orders of magnitude fewer antibodies over a longer period (Andersson, Björkelund, & Malmqvist, 2010). Furthermore, as antibody‐antigen equilibrium is more likely to be achieved with extended incubation times, this can lead to decreased variability between experiments. Indeed, we have found that overnight staining can help to circumvent many of the difficulties of high‐parameter panel optimization and is largely underutilized in flow cytometry laboratories.

NOTE: Figures throughout this text represent real data generated by our laboratory. For information regarding specific reagents and experimental conditions, please refer to the Appendix.

### Improved sensitivity can be achieved by overnight antibody staining

**Figure 1:**
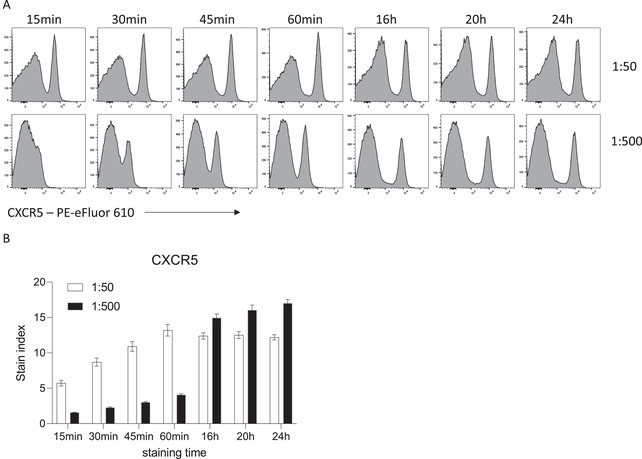
Influence of time and antibody concentration on mouse CXCR5 staining. **(A)** Representative histograms of CXCR5 staining on viable C57BL/6 mouse splenocytes after the indicated incubation times. **(B)** Stain index (n = 5, mean ± SD).

Upon the addition of fluorophore‐conjugated antibodies to a single‐cell suspension, antibody binding to the antigen of interest is rapid and can occur within minutes. However, the level of fluorescence must be sufficient to allow interpretation of antigen expression above a negative control, whether that be an internal reference population or a fluorescence‐minus‐one control. Increasing the period of incubation between an antibody and a mixed cell suspension results in an increased positive signal for a given antibody. For example, surface staining of mouse splenocytes with CXCR5‐PE‐eFluor610 at a fixed concentration leads to increased MFI as incubation is lengthened in 15‐min intervals (Fig. 1). When longer incubation times of 16 to 20 hr are used, 10‐fold less antibody is required to achieve the same MFI as in the shorter incubations.

**Figure 2:**
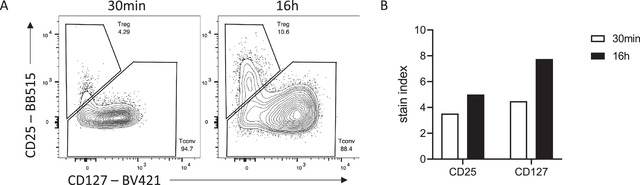
Superior discrimination of human Treg by overnight staining. **(A)** Representative flow staining of CD4^+^ CD3^+^ cells stained for 30 min or 16 hr. **(B)** Stain indices of CD25 and CD127 stained for 30 min or 16 hr. Data were acquired on a BD LSRFortessa cytometer.

Many antigens are often difficult to resolve by flow cytometry, perhaps due to the low‐affinity antibodies used to detect them. Increased incubation times can be helpful for these targets and increase their resolution beyond what is possible with standard 30‐ to 60‐min incubation times. The antibody binding rate is also limited by diffusion, that is, encountering the antigen. If the antigen is rare or hard to access, longer incubation times will facilitate binding. An example of improved resolution with longer binding can be seen when trying to detect human regulatory T cells (Treg). High expression of the high‐affinity IL‐2 receptor (CD25) paired with low expression of the IL‐7 receptor alpha (CD127) is often used to delineate Treg from conventional T cells among CD4^+^ T cells. With a 30‐min surface incubation, optimal titration can detect a CD25^hi^ CD127^lo^ Treg population (Fig. 2). However, overnight staining with a reduced concentration of antibody increases the dynamic range of both CD25 and CD127 staining, allowing for more accurate gating of the Treg population.

**Figure 3:**
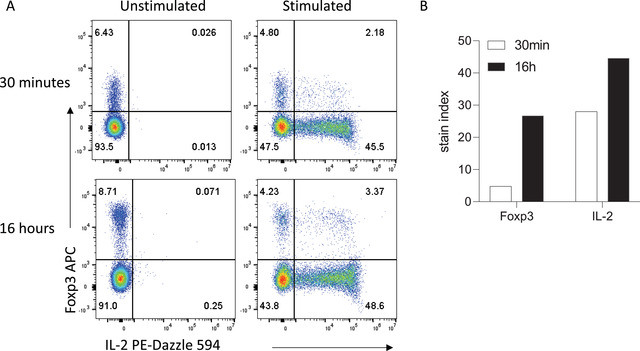
Intracellular and intranuclear staining can be improved with extended incubation times. **(A)** Representative staining obtained in 30 min versus 16 hr. **(B)** Stain indices for Foxp3 and IL‐2. Mouse splenocytes were stimulated and stained for intracellular cytokines as described in the Methods section. Data were acquired on a Cytek Aurora Spectral Cytometer.

Longer incubation times can also be utilized for intracellular detection of cytokines, transcription factors, and other intracellular proteins where access to the antigen may be restricted by cellular components. After surface staining and fixation, overnight antibody staining of permeabilized cells can lead to increased signal far beyond what is achievable with 30‐ to 60‐min incubation times, while maintaining specificity. For example, overnight staining to detect IL‐2 production by CD4^+^ T cells after *ex vivo* stimulation results in increased IL‐2 signal compared with 30‐min staining, with no signal seen in unstimulated cells in either condition (Fig. 3). Moreover, separation of the transcription factor Foxp3 is also vastly increased, allowing clear resolution between Foxp3^−^ conventional T cells and Foxp3^+^ regulatory T cells.

**Figure 4:**
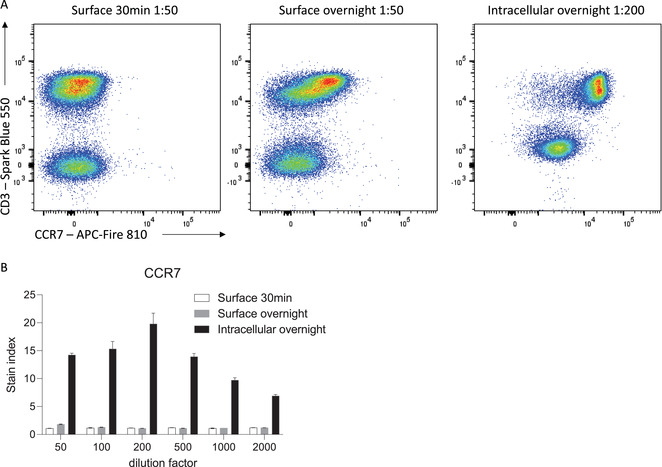
Improved detection of CCR7 with overnight intracellular staining. **(A)** Representative histograms of CCR7 staining on viable C57BL/6 mouse splenocytes after the indicated incubation times. **(B)** Stain index of CCR7 on CD3^+^ T cells (n = 3, mean ± SD).

Furthermore, overnight staining of permeabilized cells can allow for efficient detection of both surface and intracellular stores of the protein of interest. Depending on the biological question being asked, this may be advantageous in defining clear cellular populations with increased resolution. For example, many proteins are internalized upon stimulation or ligand‐binding, reducing the amount of protein available on the cell surface for antibody engagement. In scenarios where detection of surface protein specifically is not required, such as for defining cell populations based on lineage markers, fixation and permeabilization coupled with overnight staining can allow for optimal detection of protein expression. For example, the chemokine receptor CCR7, which is expressed by naïve and some memory T cells, is internalized in the presence of its ligands, CCL19 and CCL21, which are highly abundant in secondary lymphoid organs where these cells often reside (Comerford et al., 2013; Förster, Davalos‐Misslitz, & Rot, 2008). Direct surface staining of CCR7 on freshly isolated splenic T cells leads to suboptimal resolution of CCR7‐expressing cells and an underestimate of the true proportion of CCR7‐expressing cells (Fig. 4). Overnight surface staining increases this proportion; however, it is only with fixation and permeabilization to capture both surface and internalized protein that CCR7^+^ and CCR7^−^ populations are optimally resolved. Thus, optimal flow cytometry staining is highly dependent on titrated antibody concentration, incubation times, and fixatives in the context of the biological question being asked.

### Extended incubation times can reduce interexperimental variability and batch effects

**Figure 5:**
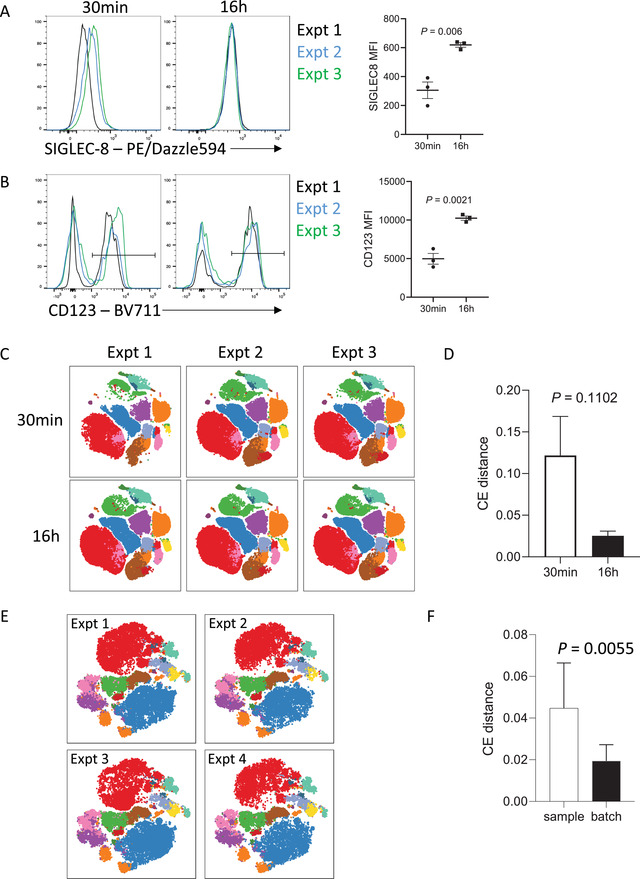
Increasing incubation time reduces batch effects. **(A)** Representative staining and MFI of SIGLEC‐8 on CD45^+^ SSC^hi^ CD16^‐^ cells from the same donor over 3 independent experiments. **(B)** Representative staining and MFI of CD123 on CD45^+^ SSC^lo^ CD3^−^ CD19^−^ CD14^−^ CD16^−^ cells from the same donor over 3 independent experiments. **(C)** Human whole blood immunophenotyping data from the same donor over three independent experiments, stained for 30 min or 16 hr. tSNE plots were generated using the parameters CD45, SSC‐A, CD4, CD8, CD127, CD16, CD19, CD3, CD123, CD20, CD25, Fcer1a, CD11c, SIGLEC‐8, CD56, CD14, and HLA‐DR. Data were acquired on a BD LSRFortessa cytometer. FlowSOM clusters are shown in a colored overlay. **(D)** Cross entropy distances between samples stained for 30 min or 16 hr. **(E)** Mouse data from four experiments over the course of two years. Data were acquired on a BD FACSymphony A5 cytometer. tSNE plots were generated using the parameters CD4, CD8, Foxp3, CD103, Neuropilin, CD44, CD62L, Ki67, ICOS, PD‐1, CTLA‐4, CD25, KLRG1, CD69, ST2, and Helios on CD3^+^ T cells. FlowSOM clusters are shown in a colored overlay. **(F)** Cross entropy distances between mouse samples (intra‐batch variation) or batches (inter‐batch variation). Significance was tested by unpaired t‐test.

A key advantage of extended antibody incubation times is that variability is often reduced between independently performed experiments. Antibody‐antigen binding is a reversible chemical reaction that initially proceeds with the rate of binding (on‐rate) exceeding the rate of dissociation (off‐rate). For short incubation times of 15 to 30 min, small differences in incubation times can translate into variable mean fluorescence intensities (MFIs), which often precludes accurate pooling of independent experiments. This is likely due to the antigen:antibody binding reaction still being within the exponential phase when the incubation stops. However, for extended incubation times of 16 to 20 hr, this variability is reduced as the antibody‐antigen complexes are closer to, if not at equilibrium. At the point of equilibrium in an antibody‐antigen interaction, the on‐rate of antibody binding is equivalent to the off‐rate and so the signal detected will be more stable. When combined with standardized sample processing methods, this extended incubation time can reduce batch effects of independent staining to be almost negligible. This is seen in Figure 5, where cryopreserved whole blood from one patient was thawed and stained on three separate occasions with a 17‐color immunophenotyping panel. The variability between experiments is evident when samples were surface stained with antibodies for 30 min (Fig. 5A and B), particularly for antigens that are more difficult to resolve, such as SIGLEC‐8. When samples were stained with the same panel, but overnight with reduced antibody, the variability between experiments was markedly diminished, in addition to the increased resolution seen. The reduction in batch effects with overnight staining was also evidenced by the reduced cross‐entropy distance between samples compared with those only stained for 30 min (Fig. 5C and D) (Roca, Burton, Neumann, et al., 2021). Similarly, variability between experiments was minimal when naïve mouse splenocytes were stained overnight with a 23‐color immunophenotyping/T cell panel on four independent occasions with different naïve mice over the course of 2 years (Fig. 5E and F). The cross‐entropy distance between batches (inter‐batch variation) was significantly lower than the cross‐entropy distance between biological replicates (intra‐batch variation), indicating that the influence of batch effects in this series of experiments was negligible. In our experience, this staining strategy has allowed for pooling of data between experiments performed by independent investigators over a year apart, with minimal variation in data quality. Overnight staining can thus be of real benefit when longitudinal analyses are required to reduce batch‐specific effects.

### Increased incubation times can reduce costs

**Figure 6:**
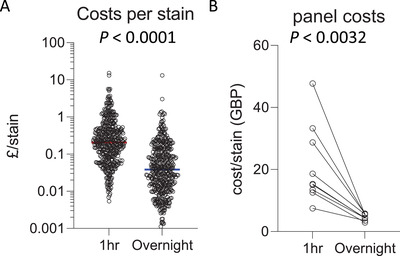
Overnight incubation increases cost‐effectiveness. **(A)** Cost (GBP) per antibody per stain for optimal titration for 1‐hr incubation (median cost £0.21) versus overnight incubation time (median cost £0.04). n = 439, Wilcoxon matched‐pairs signed‐rank test. **(B)** High‐parameter (23‐50 color) panel costs with 1 hr versus overnight incubation. Paired t‐test.

Antibody titration is a critical component of setting up a successful flow cytometry panel to achieve optimal resolution of the positive signal from negative background. With too little antibody, there will be insufficient positive signal over the background peak. With too much antibody, antibody binding to lower‐affinity targets or non‐specific binding can result in a positive shift or spread in the negative population, which also leads to difficulty in correctly interpreting positive thresholds. The optimal amount of antibody must be empirically determined for the precise conditions in which it will be used. For extended incubation times, 5‐ to 100‐fold less antibody is often required for overnight staining compared with the amount of antibody optimal for 30‐min staining. Increasing the antibody incubation period can thus be a more cost‐effective approach to high‐parameter flow cytometry. Direct comparison of the cost per stain of antibodies in use by the authors shows that the optimal antibody titration for 60‐min staining (median cost £0.21 per antibody) is significantly higher than the optimal titration for overnight staining (median cost £0.04 per antibody) (Fig. 6). Furthermore, overall costs for high‐parameter panels (23‐50 parameters) that have been titrated for overnight staining are significantly cheaper than the same panel titrated for 60‐min staining, with a median cost‐saving of £11.03 per sample. This substantial reduction in cost is an important consideration as the number of parameters increase, with 40+ fluorophores routinely used in panels with spectral flow cytometry.

### Increased flexibility in panel design

Careful panel design is an essential component of successful high‐parameter flow cytometry. In general, to decide which fluorophore conjugate to use to detect a given antigen, the properties of each antigen in the panel must be considered both in isolation and as a whole. This includes the level of expression of the antigen, as well as which antigens in the panel will be co‐expressed. Antigens expressed at low levels are generally coupled with bright fluorophores and fluorophores that will not receive high amounts of spread from fluorophores of co‐expressed markers, which can reduce resolution (Mahnke & Roederer, 2007). However, there are times during panel design when compromises must be made to balance antigen choices in the panel with antibody‐conjugate availability. Furthermore, to fully utilize the fluorescence spectrum available with spectral cytometry in order to maximize the parameters measured, antigens often have to be detected with dyes that may be sub‐optimal for their specific expression characteristics.

**Figure 7:**
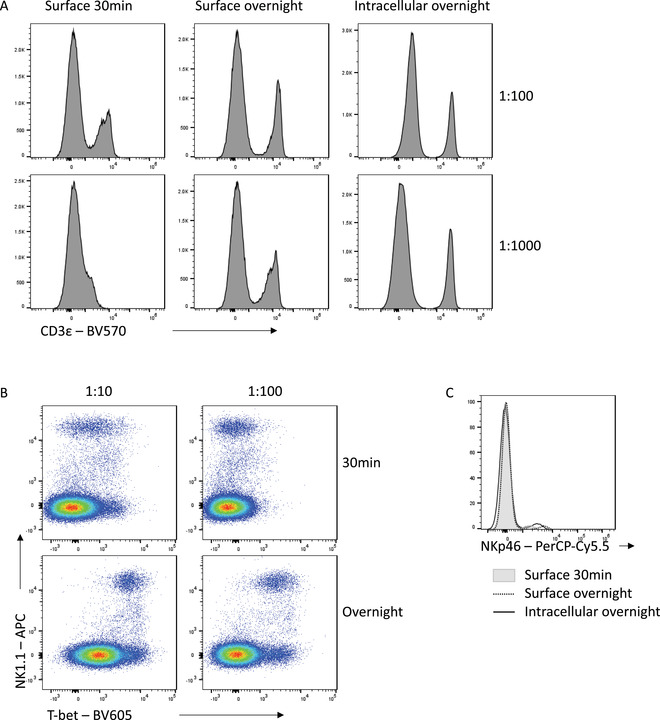
Enhanced detection of low expression markers or dim fluorophores with overnight staining. **(A)** CD3 – BV570 staining on viable splenocytes at the indicated times and dilutions. **(B)** T‐bet – BV605 staining on NK cells. **(C)** NKp46‐PerCP‐Cy5.5 staining. Examples shown are gated on viable non‐autofluorescent splenocytes.

Improving the signal:noise ratio of antibody staining by appropriately combining fixation choices and overnight staining can result in increased flexibility in panel design. With standard staining conditions, some fluorophores are simply not bright enough to be used to detect certain antigens. An example is CD3‐BV570, which leads to suboptimal resolution of CD3^−^ and CD3^+^ populations when used for surface staining for 30 min, even at high concentrations (Fig. 7A). However, overnight staining with the same antibody allows for clear separation of these populations, even when used at a 10‐fold lower concentration. An increased incubation time increases the time available for an antibody to find its antigen, which is important when using low‐affinity antibodies or when antigens are difficult to access. For example, for Tbet‐BV605, a 30‐min incubation time is insufficient to resolve expression even when highly concentrated (Fig. 7B). By instead incubating this antibody overnight, Tbet expression can be clearly resolved, while maintaining specificity. Similarly, an overnight incubation with NKp46‐PerCP‐Cy5.5 either on the surface or intracellularly increases the capacity to detect the NKp46^+^ population in mouse splenocytes relative to a 30‐min incubation time (Fig. 7C). The improved staining seen with extended incubation times therefore allows for incorporation of antibody‐fluorophore choices into panels which would otherwise have insufficient resolution to be a useful option.

### Reduced interference from unwanted polymer dye and fluorophore interactions

**Figure 8:**
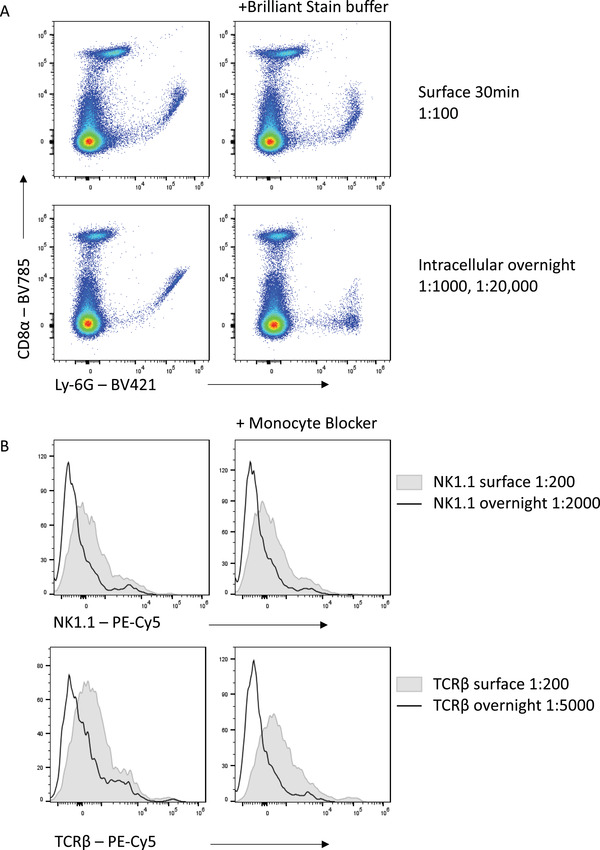
Reduced non‐specific binding with lower concentrations of antibody in overnight staining. **(A)** Brilliant Violet dye interactions in a 30 min stain versus an overnight stain. **(B)** Non‐specific binding of PE‐Cy5 tandems to macrophages in 30 min versus overnight surface staining. Histograms shown are gated on viable F4/80^+^ autofluorescent macrophages.

In addition to minimizing cost, the reduced antibody concentration generally required when incubation times are increased has other benefits. With panels containing large numbers of polymer dyes (including Brilliant Violet, Brilliant Blue, and Brilliant Ultraviolet reagents), aggregation of different polymer dyes can skew fluorescence signals and lead to misinterpretation of expression data. This has necessitated the development of buffers such as Brilliant Stain Buffer (BD Biosciences) and Super Bright Complete Staining Buffer (Thermo Fisher) to limit polymer dye interactions. As the interaction between dyes is proportional to the antibody concentration, limiting the antibody used can also lead to reduced polymer aggregation and thus cleaner, more interpretable data (Fig. 8A). This is particularly important for high‐parameter panels where many polymer‐based reagents are combined.

Other instances of non‐specific binding also complicate high‐parameter flow, with certain fluorophores capable of binding directly to Fc receptors or other surface molecules (Jahrsdörfer, Blackwell, & Weiner, 2005; Park, Rodriguez, & Steinman, 2012). This is particularly a problem for tandem dyes containing the cyanine acceptor such as Cy5, which bind to the high‐affinity IgG receptor CD64 (van Vugt, van den Herik‐Oudijk, & van de Winkle, 1996). Thus, using these dyes for CD64‐expressing cells, such as macrophages, is problematic and leads to non‐specific signal. This can be reduced by the addition of phosphorothioate oligodeoxynucleotides (Jahrsdörfer et al., 2005) or commercial blocking reagents, such as the True‐Stain Monocyte Blocker (Biolegend) (Fig. 8B). However, substantial reduction in non‐specific fluorophore binding can be achieved with the reduced concentration of Cy5‐tandem dyes needed when staining overnight compared with 30‐min staining.

## PRACTICAL CONSIDERATIONS IN OPTIMIZING STAINING CONDITIONS

### Optimizing staining conditions and antibody titration

After carefully designing your flow cytometry panel, the next step is to test and optimize the panel to ensure that each marker is sufficiently resolved. The first component of this is to titrate each antibody to find the optimal concentration in which clear positive signals can be obtained. Each antibody must be titrated for the precise conditions in which it will be used, as incubation time, temperature, and sample characteristics (such as cellular origin and processing conditions) can all affect how much antibody is required for optimal separation.

**Figure 9:**
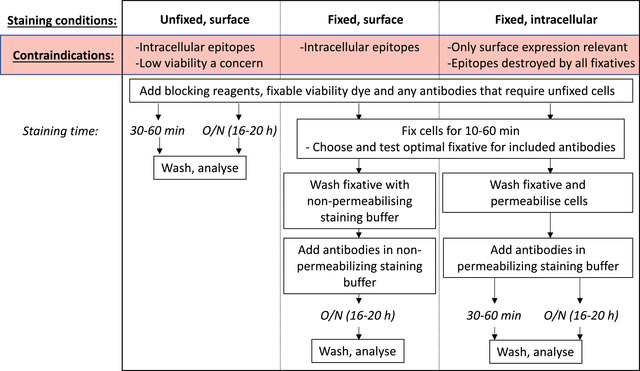
Protocol overview for optimizing staining conditions.

Because of the generally improved resolution seen with overnight antibody incubation, we recommend as a starting point to titrate each antibody for a 30‐ to 60‐min surface staining, overnight surface staining, and overnight intracellular staining (Fig. 9). However, this clearly must be modified to be within the bounds of the biological context of each experiment. For some experiments, intracellular staining of a protein is not desirable because cell surface expression is the desired biological readout. For cytoplasmic or nuclear proteins, fixation and permeabilization are essential. In experiments where viability is of particular concern, overnight surface staining of unfixed cells may not be a useful option, as the prolonged incubation may have too much impact on cell viability (discussed later). Once the possible experimental conditions have been determined, the antibody must be titrated individually for each of those conditions. Where the manufacturer has given recommendations on the amount of antibody to use per test, this is generally a good starting point for the highest concentration of antibody to use, followed by 2‐fold dilutions. It is essential to include a viability dye to remove non‐specific signal by dying or dead cells. If the antibody being titrated recognizes an epitope that is only expressed on a rare population, you may also need to include a fixed concentration of other antibodies to define this lineage and enable proper analysis of the signal being titrated. Once tested, the optimal staining conditions for each antibody can be determined by analyzing the staining index (Maecker, Frey, Nomura, & Trotter, 2004):

$$Staining\;index=\left(\frac{MFI_{positive}-MFI_{negative}}{2\times SD_{negative}}\right)$$

The staining index is a useful tool because it accounts for both the magnitude of the positive signal achieved from antibody binding to the antigen (positive), as well as the background signal on the population not expressing the antigen of interest (negative).

**Figure 10:**
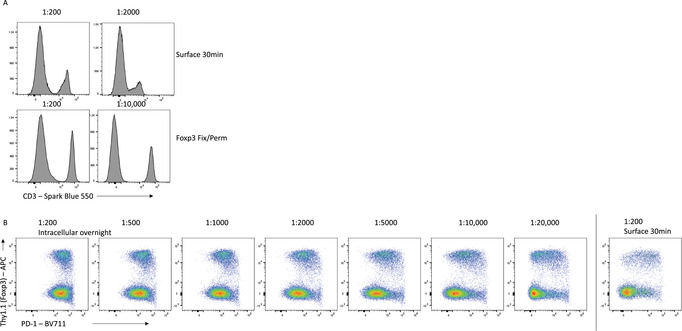
Titration is essential to maximize sensitivity. **(A)** Titration of CD3‐Spark Blue 550. **(B)** Intracellular overnight staining for PD‐1 on viable CD4^+^CD3^+^ T cells at the indicated dilutions on cells fixed and permeabilized with the eBioscience Foxp3 Fix/Perm kit.

Titration is absolutely essential to maximize the resolution of each antibody, and different staining conditions can influence the optimal amount of antibody required. For example, staining of mouse splenocytes with CD3‐SparkBlue550 on unfixed cells for 30 min requires an optimal dilution of 1:200 (Fig. 10A). However, for fixed cells stained overnight with the same antibody, this dilution leads to expanded background signal and poorer resolution of the CD3‐expressing cells, and a 1:10,000 dilution is optimal. Similarly, titration of PD‐1‐BV711 on mouse CD4^+^ T cells shows that using too high concentrations of antibody increases the background signal on PD‐1‐negative cells to the point where a distinction in expression cannot be determined (Fig. 10B). Proper titration for each of the staining conditions reveals that 100‐fold less PD‐1 antibody is required when staining intracellularly overnight, compared with surface staining for 30 min.

### Choice of fixative and effects on epitopes

**Figure 11:**
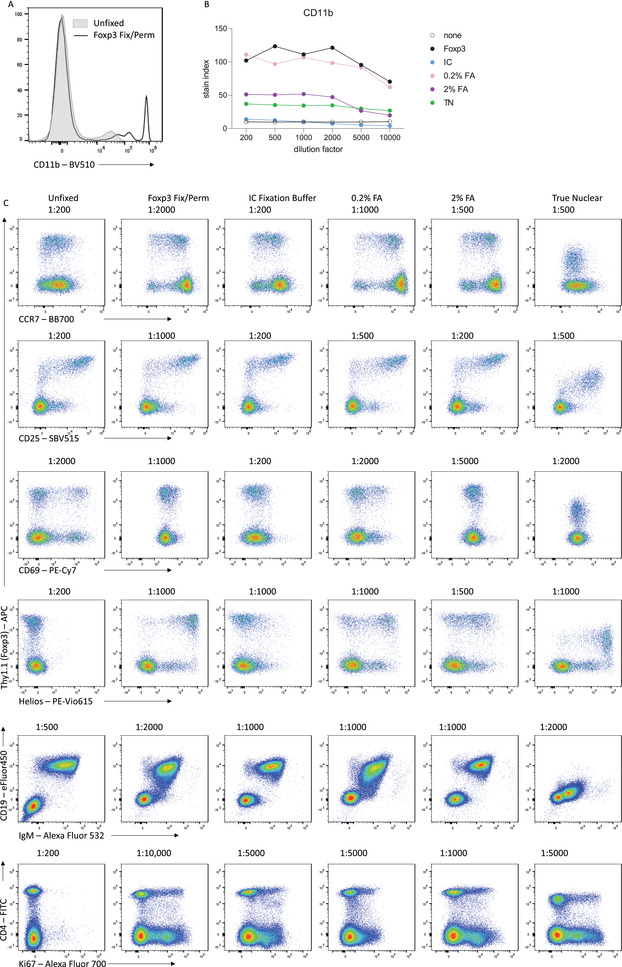
Choice of fixative affects staining intensity and specificity. **(A)** Representative histograms showing overnight CD11b‐BV510 staining on mouse splenocytes either with or without fixation. **(B)** Stain indices for CD11b staining at various dilutions with various fixatives. **(C)** Representative examples of overnight marker staining on murine splenocytes with various fixatives.

In many cases, antibody staining after fixation and permeabilization will lead to improved staining sensitivity. However, the type of fixative used can dramatically impact how well the antibody will bind its antigen. The most commonly used fixatives for flow cytometry are formaldehyde‐based, with an active concentration of 1% to 4% formaldehyde. Formaldehyde reacts with amino acids, linking adjacent proteins into a rigid matrix (Kamps, Hopkinson, Schofield, & Claridge, 2019). This preserves the cellular structure but may also impact the ability of the antibody to recognize the epitope if it has been altered by the chemical reaction. Commercially available fixatives or kits may also include methanol or detergents such as Triton X‐100 or saponin to assist with permeabilization. These permeabilizing agents remove lipids or cholesterol molecules, creating holes that allow high‐molecular‐weight antibody:fluorophore conjugates to pass through the plasma, organelle, and nuclear membranes. The type of fixative, as well as the fixation conditions, can dramatically impact how well an antibody will be able to bind its antigen. In Figure 11, we compare antibody staining on mouse splenocytes for commonly used fixatives: the eBioscience Foxp3/Transcription Factor Fixation Buffer (Foxp3 Fix/Perm; ThermoFisher), the eBioscience IC Fixation Buffer (ThermoFisher), a 0.2% formaldehyde solution (as formalin), a 2% formaldehyde solution, and the True‐Nuclear Transcription Factor Buffer Set (True Nuclear Fix; BioLegend).

Many antibodies, including CD11b‐BV510 and CCR7‐BB700, have improved sensitivity when used on fixed cells compared with unfixed surface staining. CD25‐SBV515 stains well under all fixation conditions, although reduced concentrations are required when using certain fixatives such as Foxp3 Fix/Perm. For an antigen such as CD69, optimal staining is achieved in the absence of fixation, but is still resolvable after a light fixation with 0.2% formaldehyde solution. After stronger fixing with eBioscience Foxp3 Fix or 2% formaldehyde, the ability of the antibody to bind its epitope is lost. For the transcription factor Helios, optimal staining is achieved after fixation with eBioscience Foxp3 Fix/Perm and True Nuclear Fix, which have been optimized for the detection of intranuclear proteins, whereas fixation with formalin‐only solutions leads to suboptimal detection of Helios after permeabilization. The preservation and accessibility of each epitope after different fixation methods must be empirically determined, although online resources are available for the commonly used antibody clones. We find that the eBioscience Foxp3 Fix/Perm regents provide a good balance between preservation and accessibility for most murine targets.

Another advantage of fixation is that it can allow for the co‐detection of fluorescent reporter proteins, such as GFP and RFP, with intracellular proteins such as cytokines or transcription factors. Fixation needs to be sufficiently strong to retain the fluorescent proteins within the cell prior to permeabilization, such as with a 2% formaldehyde solution, whereas fixation with Foxp3 Fix/Perm leads to leakage from the cell and subsequent loss of signal (Heinen et al., 2014). The concentration and fixative incubation time will alter the subsequent detection of intracellular or intranuclear proteins, and needs to be optimized for the antigens of interest. Further flexibility can be achieved by recovering fluorescent signal with antibodies directed toward the fluorescent protein, with anti‐GFP and RFP antibodies readily available commercially.

In designing a staining strategy, a further consideration is which antibodies need to be stained prior to fixation, as the fluorophores used can be altered by different fixatives. In general, tandem dyes are particularly susceptible to fixation. Fixation can induce degradation of the tandems and subsequent loss of signal, as well as create a false positive signal in the donor fluorophore of the tandem. Where possible, it can be advantageous to use these sensitive fluorophores to detect antigens post‐fixation.

### Controlling for non‐specific staining

**Figure 12:**
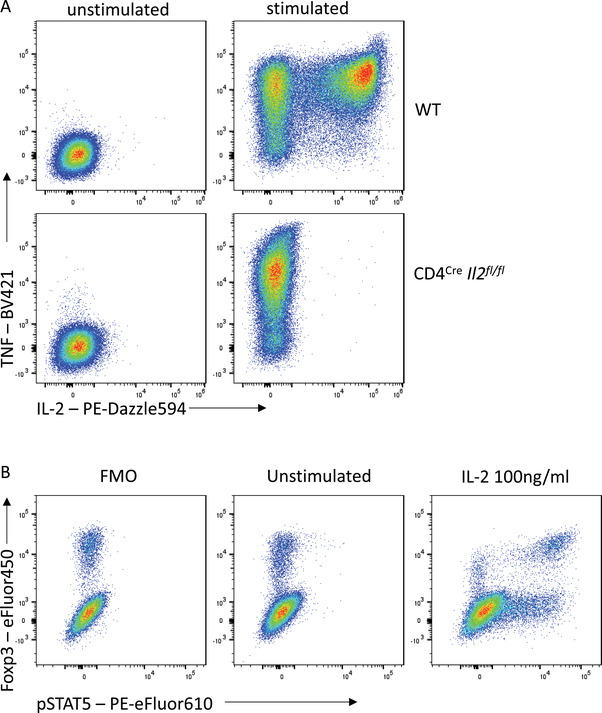
Controls confirm specificity is maintained with overnight staining. **(A)** IL‐2 staining on WT or IL‐2‐deficient mouse CD4^+^ T cells. **(B)** pSTAT5 and Foxp3 staining on mouse CD4^+^ T cells with or without IL‐2 stimulation.

A misconception regarding extended incubation times is that the increased signal intensity must be the result of non‐specific staining. While this can certainly be the case, particularly when antibodies have not been titrated for the conditions, non‐specific binding can affect all flow cytometry staining and should be controlled for wherever possible. The ideal controls to test for non‐specific staining of an antibody on a specific cell type are cells with similar autofluorescence profiles that are known to not express the antigen recognized by the antibody. The gold standard is therefore to stain cells prepared identically but which lack the antigen of interest due to genetic deficiency, i.e., comparing wild‐type and knock‐out cells (Fig. 12A). This is often not feasible, so another useful option can be an internal negative, which is a cell type within the stained sample that is known empirically to not express the antigen. An advantage of this control is that because the cell types are within the same sample, the staining conditions are identical. However, for internal negative controls, it is critical that the cell types being compared have similar autofluorescence profiles, as this can dramatically change the staining profile. For example, granulocytes generally exhibit significantly higher background signals than lymphocytes as a result of their increased granularity and distinct cellular contents. For this reason, gates designated based on the background signal in T cells will not be suitable for setting gates for eosinophils.

Moreover, even cell types with similar autofluorescence profiles can have different background signals in particular detectors as a result of the spectral spread of other fluorophores used in the panel. Fluorescence‐minus‐one (FMO) controls are thus essential to appropriately interpreting the influence of spectral spread on your staining. Lastly, when testing for stimulation‐induced signals, such as cytokine release or phosphorylated proteins, an unstimulated control can be a useful biological control (Fig. 12B).

### When overnight surface staining is sometimes preferable: effects on staining and viability

There are circumstances in which overnight staining of unfixed cells will give optimal results. Aside from situations where surface expression is the desired biological readout, detection of some proteins is simply better with surface staining or may lead to non‐specific binding when the same antigen is stained intracellularly. One such example is the chemokine receptor CXCR5, which has a vastly improved signal‐to‐noise ratio across varied titrations with overnight surface staining, compared to a standard surface stain or an overnight intracellular stain (Fig. 1 and data not shown). Human samples can also often have an increased background when antigens are stained intracellularly.

**Figure 13:**
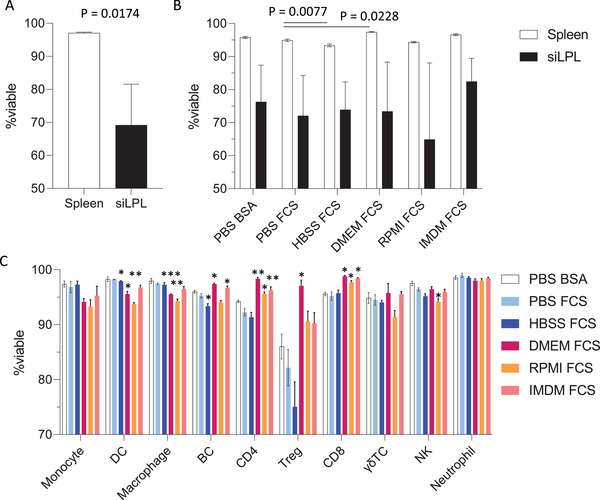
Buffer composition and preparation viability affects cell survival during overnight incubation. **(A)** Leukocyte (CD45^+^) viability prior to overnight incubation (n = 3, mean ± SD) in mouse spleen or small intestinal lamina propria leukocytes. Statistical analysis by unpaired t‐test. **(B)** Impact of buffer choice on leukocyte viability in overnight incubation. Cellular viability was assessed on single CD45^+^ leukocytes that were negative for fixable viability dye prior to overnight staining. **(C)** Viability of various immune cell types from mouse spleen after overnight incubation in different buffers. Statistical analysis for B and C by 2‐way ANOVA with Dunnett's multiple comparisons between PBS FCS and all other conditions.

To decide whether overnight staining of unfixed cells is the right choice for your experiment, a key consideration is viability. For cells that are generally in good condition, such as freshly isolated mouse splenocytes, overnight staining of healthy cells only results in a ∼5% loss of overall viability (Fig. 13). In this setting, viability remains above 90% in most cell types, dropping the most for Treg at ∼80% viability when stained in PBS‐FCS‐EDTA (Fig. 13C). Cells that are more fragile, such as those that have been cryopreserved or obtained from tissues after lengthy digestion times, may be more sensitive to overnight incubation and thus fixing the cells prior to incubation may be a more appropriate strategy. Leukocytes isolated from murine small intestine can have impaired viability due to the lengthy processing procedure (Fig. 13A), and the extended incubation time for flow staining results in a further loss of viability (Fig. 13B). The effects on viability for your cell types of interest should be determined empirically for your staining conditions. In many cases, a negligible or slight loss in viability is an acceptable compromise for the improved staining resolution gained with the extended incubation time.

The choice of staining buffer can also impact cell viability. In our hands, staining cells with a complete medium such as IMDM or RPMI can significantly improve viability compared with staining in PBS or HBSS. This is particularly pronounced for more sensitive cells such as Tregs, where incubation in complete medium maintains viability at 90% or higher (Fig. 13C). A protein source such as fetal bovine serum (FBS) or bovine serum albumin (BSA) is generally included in staining buffers to reduce non‐specific binding, although this choice did not appear to significantly impact cell viability.

### Convenience and working conditions

Researchers using an overnight staining protocol may experience additional benefits from this approach due to the way this method breaks up the experiment over two days. For larger experiments, sample preparation may take up the bulk of the working day, leaving the researcher to compete for limited hours on busy cytometers in the afternoon, or run late into the evening. Longer days are more likely to result in rushed acquisition or mistakes due to fatigue. By leaving the cells to stain overnight and returning to acquire the following morning, researchers may find that they have little to no competition for the cytometer of their choice, and that their results are less affected by machine issues. For shared resource labs, distributing the instrument use across the entire day will make better use of the machines and potentially provide more revenue.

### CONCLUDING REMARKS

High‐parameter flow cytometry is an integral technique for biological interrogation in diverse applications. Each antigen to be recognized in flow cytometry has distinct characteristics in terms of the amount and location of expression, and the antibodies used to detect these antigens also vary in their affinity, brightness, and binding features. Despite this, standard flow cytometry methods often use generic, relatively short staining conditions to detect all of these diverse conditions. This often results in substandard staining, with difficulties in correctly identifying positive expression from background levels of fluorescence. Vastly improved resolution can be achieved by optimizing staining conditions for each antigen, enabling more accurate quantitation of marker expression. These optimized conditions also have flow‐on effects to reduce experimental costs and enable increased flexibility in panel design. Taking an extra day to optimize staining conditions for your antigens of interest can thus pay dividends in achieving cheaper and more accurate flow data.

### Author Contributions

**Carly Whyte**: Conceptualization, Formal analysis, Investigation, Methodology, Visualization, Original draft; **Damon Tumes**: Funding acquisition, Draft review and editing; **Adrian Liston**: Funding acquisition, Draft review and editing; **Oliver Burton**: Conceptualization, Formal analysis, Investigation, Methodology, Visualization, Original draft.

### Conflict of Interest

O. Burton has consulted for Bio‐Rad regarding Star Bright dyes. C. Whyte, D. Tumes, and A. Liston have no conflicts of interest to disclose.

## Data Availability

The data that support the findings of this study are available from the corresponding author upon reasonable request.

## Appendix

Cells were routinely counted using an automated cell counter (Countess II, ThermoFisher) or by manual counting, and two million cells were used for each stain. Cells were routinely stained in PBS with 2.5% FCS (S00115 Tico Europe) and 2 mM EDTA (15575020 ThermoFisher), referred to as FACS buffer. Additional buffers used include HBSS (14025092, ThermoFisher), DMEM (10564011, ThermoFisher), and RPMI (21875091, ThermoFisher). The 2.4G2 hybridoma supernatant (anti‐mouse CD16/32) was prepared in‐house in DMEM and used routinely for blocking for at least 30 min prior to staining murine cells. Human BD Fc Block (564220, BD Biosciences) was used routinely for blocking for at least 30 min prior to staining human cells. The antibodies used are listed in Table 1. Additional reagents include Brilliant Stain Buffer (563794, BD Biosciences), True‐Stain Monocyte Blocker (426103, BioLegend), and BSA (126609‐5GM, Sigma‐Aldrich).

**Table 1:** Reagents Used for This Study

Antibody	Clone	Concentration	Figures	Company	Catalog #
Anti‐mouse CXCR5 PE‐eFluor610	SPRCL5	1:50, 1:500	1	ThermoFisher	61‐7185‐82
Fixable viability dye eFluor 780	N/A	1:4000	1‐5, 7‐13	ThermoFisher	65‐0865‐18
Anti‐human CD25	BC96	1:100, 1:200	2, 5	BD Biosciences	567318
Anti‐human CD127	HIL‐7R‐M21	1:50, 1:100	2, 5	BD Biosciences	562436
Anti‐human CD3	UCHT1	1:50, 1:100	2, 5	BD Biosciences	563852
Anti‐human CD4	SK3	1:133, 1:250	2, 5	BD Biosciences	612937
Anti‐mouse IL‐2 PE‐Dazzle 594	JES6‐5H4	1:250	3, 12	BioLegend	503840
Anti‐mouse Foxp3 APC	FJK‐16s	1:200	3	ThermoFisher	17‐5773‐82
Anti‐mouse CD4 Brilliant Violet 421	GK1.5	1:1000	3	BioLegend	100443
Anti‐mouse CD3 Alexa Fluor 488	145‐2C11	1:1000	3	BioLegend	100321
Anti‐mouse CCR7 APC‐Fire 810	4B12	1:50, 1:200	4	BioLegend	120135
Anti‐mouse CD3 Spark Blue 550	17A2	1:500, 5000, 1:200, 1:10,000	4, 10, 13	BioLegend	100260
Anti‐mouse CD69 PE‐Cy5	H1.2F3	1:200	5	BioLegend	104510
Anti‐mouse CD25 Brilliant Violet 480	PC61	1:1000	5	BD Biosciences	566120
Anti‐mouse CD103 Brilliant Ultraviolet 395	M290	1:500	5	BD Biosciences	740238
Anti‐mouse CD4 Brilliant Ultraviolet 496	GK1.5	1:500	5, 13	BD Biosciences	612952
Anti‐mouse CD152 (CTLA‐4) Brilliant Violet 421	UC10‐4B9	1:200	5	BioLegend	106312
Anti‐mouse CD45 Brilliant Ultraviolet 563	30‐F11	1:10,000	5	BD Biosciences	565710
Anti‐mouse CD62L Brilliant Ultraviolet 737	MEL‐14	1:1000	5	BD Biosciences	612833
Anti‐mouse CD8a Brilliant Ultraviolet 805	53‐6.7	1:1000	5, 13	BD Biosciences	564920
Anti‐mouse CD44 Brilliant Violet 570	IM7	1:500	5	BioLegend	103037
Anti‐mouse CD278 (ICOS) Brilliant Violet 605	C398.4A	1:500	5	BioLegend	313538
Anti‐mouse CD3 Brilliant Violet 650	17A2	1:2000	5	BioLegend	100229
Anti‐mouse PD‐1 Brilliant Violet 711	29F.1A12	1:5000	5, 9	BioLegend	135231
Anti‐mouse CD19 Brilliant Violet 750	1D3	1:2000	5	BD Biosciences	Custom reagent
Anti‐mouse KLRG1 Brilliant Violet 785	2F1	1:1000	5	BioLegend	138429
Anti‐mouse CD45.1 Alexa Fluor 488	A20	1:2000	5	BioLegend	110718
Anti‐mouse CD304 (Neuropilin) PerCP‐eFluor 710	3DS304M	1:5000	5	ThermoFisher	46‐3041‐82
Anti‐T‐bet PE	REA102	1:200	5	Miltenyi Biotec	130‐098‐596
Anti‐mouse Helios PE‐Vio615	REA829	1:500	5, 10	Miltenyi Biotec	130‐112‐636
Anti‐mouse NK1.1 PE‐Cy5.5	PD136	1:10,000	5	BioLegend	108702
Anti‐mouse ST2 PE‐Cy7	RMST2‐2	1:5000	5	ThermoFisher	25‐9335‐82
Anti‐mouse Foxp3 APC	REA788	1:200	5	Miltenyi Biotec	130‐111‐601
Anti‐mouse Ki67 Alexa Fluor 700	16A8	1:1000	5	BioLegend	652420
anti‐human CD45 BUV395	HI30	1:80, 1:160	5	BD Biosciences	563792
anti‐human CD8 BUV737	RPA‐T8	1:200, 1:400	5	BD Biosciences	749367
anti‐human CD16 BV480	3G8	1:200, 1:400	5	BD Biosciences	566171
anti‐human CD19 BV605	SJ25C1	1:80, 1:160	5	BD Biosciences	562653
anti‐human CD123 BV711	9F5	1:150, 1:300	5	BD Biosciences	563161
anti‐human CD20 BV786	L27	1:80, 1:160	5	BD Biosciences	740838
anti‐human Fcεr1α PerCP‐Cy5.5	AER‐37	1:80, 1:160	5	BioLegend	334621
anti‐human CD11c PE	B‐ly6	1:20, 1:40	5	BD Biosciences	555392
anti‐human Siglec8 PE‐Dazzle594	7C9	1:150, 1:300	5	BioLegend	347110
anti‐human CD56 PE Cy7	B159	1:100, 1:200	5	BD Biosciences	557747
anti‐human CD14 AF647	MφP9	1:150, 1:300	5	BD Biosciences	562690
anti‐human HLA‐DR R718	G46‐6	1:100, 1:200	5	BD Biosciences	567035
Anti‐mouse CD3 BV570	17A2	1:100, 1:1000	7	BioLegend	100225
Anti‐mouse Ly‐6G Brilliant Violet 421	1A8	1:100, 1:20,000	8	BioLegend	127627
Anti‐mouse CD8a Brilliant Violet 785	53‐6.7	1:100, 1:1000	8	BioLegend	100750
Anti‐mouse TCRγδ PE‐Cy5	GL3	1:500, 1:2000	8	ThermoFisher	15‐5711‐82
Anti‐mouse NK1.1 PE‐Cy5	PK136	1:200, 1:2000	8, 13	BioLegend	108716
Anti‐mouse F4/80 Alexa Fluor 561	BM8	1:200	8, 13	ThermoFisher	505‐4801‐82
Anti‐mouse CD19 eFluor 450	1D3	1:1000	8, 13	ThermoFisher	48‐0193‐82
Anti‐mouse CD3 Alexa Fluor 488	145‐2C11	1:1000	8	BioLegend	100321
Anti‐CD69 PE‐Cy7	H1.2F3	1:200, 1:1000	10	ThermoFisher	25‐0691‐82
Anti‐mouse CD11b Brilliant Violet 510	M1/70	1:100, 1:1000	10, 13	BioLegend	101263
Anti‐mouse Foxp3 eFluor 450	FJK‐16s	1:200	12	ThermoFisher	48‐5773‐82
Anti‐ Phospho‐STAT5 (Tyr694) PE‐eFluor 610	SRBCZX	1:200	12	ThermoFisher	61‐9010‐42
Anti‐mouse TNF Brilliant Violet 421	MP6‐XT22	1:250	12	BioLegend	506328
Anti‐mouse Ly‐6C/Ly‐6G Alexa Fluor 532	RB6‐8C5	1:1000	13	ThermoFisher	58‐5931‐82
Fixable viability dye eFluor 506	N/A	1:500	13	ThermoFisher	65‐0866‐18
Anti‐mouse Thy1.1 APC	HIS51	1:500	13	ThermoFisher	17‐0900‐82
Anti‐mouse TCRγδ Super Bright 780	GL3	1:200	13	ThermoFisher	78‐5711‐82
Anti‐mouse I‐1/I‐E Alexa Fluor 700	M5/114.15.2	1:500	13	ThermoFisher	56‐5321‐82
Anti‐mouse CD11c Alexa Fluor 488	N418	1:1000	13	ThermoFisher	53‐0114‐82

For fixative comparisons, the following reagents were used: formalin (9713.5000, VWR), eBioscience Foxp3/Transcription Factor Staining Buffer Set (00‐5523‐00, ThermoFisher), IC Fixation Buffer (FB001, ThermoFisher), or True‐Nuclear Transcription Factor Buffer Set (424401, BioLegend).

Anti‐mouse NK1.1 was conjugated to PE‐Cy5.5 using a Lightning Link kit (Abcam, ab102899) according to the manufacturer's instructions.

For phospho‐STAT staining, cells were stained for certain surface epitopes prior to stimulation or fixation. The cells were stimulated with recombinant mouse IL‐2 (75406, BioLegend) for 20 min at 37°C in IMDM supplemented with 10% FCS. Without washing or manipulation, the cells were fixed in 4% formaldehyde for 30 min at room temperature. The cells were centrifuged and resuspended in ice‐cold methanol, mixed, and incubated on ice for 30 min. After washing in TBS with 2.5% FCS and 2 mM EDTA, the cells were stained overnight at room temperature for pSTAT5 and Foxp3. The cells were washed again twice prior to acquisition.

For intracellular cytokine staining, cells were stimulated for 4 hr at 37°C in IMDM supplemented with 10% FCS and the following mitogens: 500 ng/ml phorbol 12,13‐dibutyrate (4153 Tocris Bioscience), 750 ng/ml ionomycin (1704 Tocris Bioscience), and 1 µg/ml brefeldin A (1231 Tocris Bioscience). The cells were surface stained for 1 hr in the presence of brefeldin A at 4°C, washed, fixed for 30 min with 2% formaldehyde at room temperature, and washed twice with 1× permeabilization buffer (00‐5523‐00 ThermoFisher) prior to overnight staining for IL‐2 and Foxp3 in permeabilization buffer with 20% 2.4G2 supernatant at 4°C. The following morning, the cells were washed twice with permeabilization buffer and once with FACS buffer prior to acquisition on a Cytek Aurora.

C57BL/6 mice were used for all murine experiments. Mice were bred, housed, and culled in accordance with ethical guidelines under specific pathogen‐free conditions in facilities either at KU‐Leuven in Leuven, Belgium (Fig. 5) or the Babraham Institute in Cambridge, UK.

Gut leukocytes were isolated from the lamina propria of mouse small intestine as follows. Chyme was flushed out of the intestinal lumen and Peyer's patches were removed. The intestine was cut longitudinally, chopped into ∼1‐cm pieces, and agitated at 37°C in HBSS with 10 mM EDTA and 2.5% FCS for two rounds of 20 min to remove the epithelial layer. The pieces were then chopped finely with a razor blade and digested in IMDM with 20% FCS and 2 mg/ml collagenase IV (ThermoFisher, 17104019) for 30 min at 37°C with agitation. The remaining pieces were triturated with a pipette and filtered through 100‐µm nylon mesh. The resulting single‐cell suspension was centrifuged through 40% Percoll (Sigma Aldrich, GE17‐0891‐01) to recover leukocytes.

Whole blood was collected from human volunteers into BD Vacutainer Lithium Heparin Tubes and red blood cells were lysed before being cryopreserved. This work was done as part of study 14536 approved by the Central Adelaide Local Health Network Human Research Ethics Committee.

Statistical tests were conducted in GraphPad Prism 9.2.0. Specific tests used are indicated in the figure legends. In some cases, *p* values are abbreviated as **p* < 0.05, ***p* < 0.01, ****p* < 0.001.
